# Diagnostic Bedside Vestibuloocular Reflex Evaluation in the Setting of a False Negative Fistula Test in Cholesteatoma of the Middle Ear

**DOI:** 10.1155/2017/2919463

**Published:** 2017-03-13

**Authors:** Ricardo D'Albora, Ligia Silveira, Sergio Carmona, Nicolas Perez-Fernandez

**Affiliations:** ^1^Department of Otorhinolaryngology, Hospital de Clínicas, Facultad de Medicina UDELAR, Montevideo, Uruguay; ^2^Department of Neuro-Otology, Instituto de Neurociencias de Buenos Aires (INEBA), Buenos Aires, Argentina; ^3^Department of Otorhinolaryngology, Clínica Universidad de Navarra, Pamplona, Spain

## Abstract

*Background.* False negative fistula testing in patients with chronic suppurative otitis media is a dilemma when proceeding to surgery. It is imperative to rule out a dead labyrinth or a mass effect secondary to the cholesteatoma in an otherwise normally functioning inner ear. We present a case series of three patients in whom a bedside vestibuloocular reflex (VOR) evaluation using a head impulse test was used successfully for further evaluation prior to surgery.* Results.* In all three cases with a false negative fistula test we were able to further evaluate at the bedside and were not only able to register the abnormal VOR but also localize its deterioration to a particular semicircular canal eroded by the fistula.* Conclusion.* Vestibuloocular reflex evaluation is mandatory in patients with suspected labyrinthine fistula due to cholesteatoma of the middle ear before proceeding to surgery. We demonstrate successful use of a bedside head impulse test for further evaluation prior to surgery in patients with false negative fistula test.

## 1. Introduction

The fistula test (FT) is a bedside vestibular method of examination that detects the existence of an abnormal communication between the middle and inner ear. As described by Lucae in 1881, when applying pressure in the external ear, vertigo and nystagmus are elicited and remain for a period of time [1].

Fistula in the walls of the inner ear in cholesteatoma are linked to several factors: aggressiveness of the disease (in particular because of the matrix producing metalloproteinases, their specific inhibitors, and epidermal growth factors); its location in the middle ear space; clinical course; and treatment during its growth [2]. In patients with chronic suppurative otitis media with cholesteatoma a labyrinthine fistula has been confirmed in 3–13% of patients that underwent a surgical procedure [3].

The FT, although easy to perform and score [4], has not been found to be very sensitive, with only 20% of patients with a surgically confirmed fistula having a positive test [5]. In the case of a negative test, a false negative result has to be considered secondary to the cholesteatoma covering the fistula and exerting a mass effect preventing the transmission of pressure modifications from the external/middle ear to the inner ear or because of a dead labyrinth. Differentiating between these diagnoses is critical to the surgical approach.

Due to the inherent pathological changes in the external and middle ear of patients with cholesteatoma, a vestibular evaluation should be performed even though 53% of these patients have suffered some degree of dizziness on review of systems. In these patients, the most frequent abnormal test result was found in the rotatory chair [6]; interestingly, findings in the rotatory chair test correlate well with the caloric test performed with air stimulation [7].

Recently, new developments have provided a better insight to the pathophysiology of vestibular disorders, in particular in patients not easily evaluated by conventional methods. With the video head impulse test (vHIT) it is possible to evaluate peripheral vestibular function using proper stimulation (head velocity) in the plane of the canal that houses the receptor of interest. The response in initial eye velocity to the head impulse (<150 ms) is a specific test for the vestibuloocular reflex that is not influenced by pathological changes to the external or middle ear.

The purpose of this case series is to demonstrate a specific methodology for the evaluation of the inner ear in cases with a false negative fistula test in patients with chronic ear disease with cholesteatoma.

## 2. Case Reports

In Table 1 we present a summary of our findings using the bedside head impulse test further detailed below.

**Figure 1:**
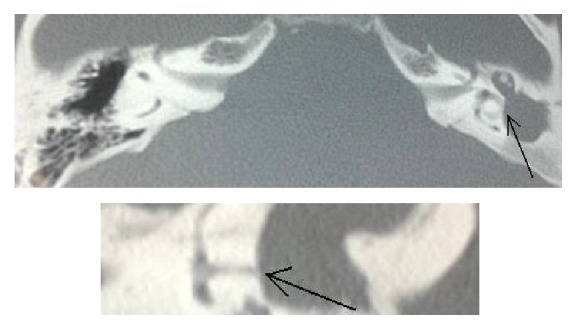
Case 1. Two different sections in the CT scan showing the fistula of the left horizontal semicircular canal and the middle ear filled with cholesteatoma and inflammatory tissue.

*Patient 1.* A 25-year-old man presented to the emergency room with an intracranial complication secondary to the chronic otitis media as shown in a CT scan (Figure 1). He was diagnosed and treated when neurologically stable. In this case the vestibular damage has occurred progressively over many years and as such was successfully compensated. The affected ear was the left; the fistula sign was negative (no nystagmus under rubber bulb applied pressure to the external ear) but the bedside head impulse test was positive due to the appearance of intense refixation saccades that indicated a major vestibular deficit.

**Figure 2:**
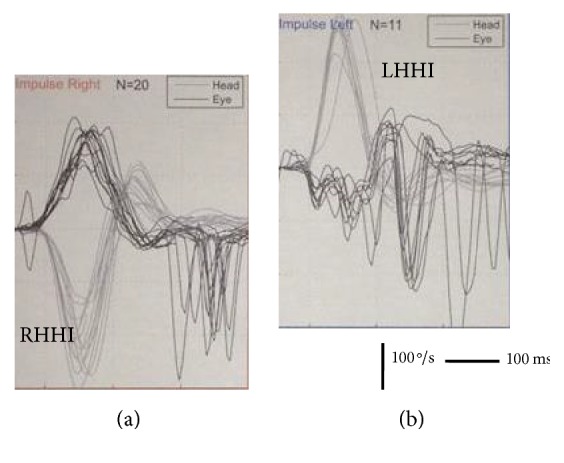
Case 2. Video head impulse test of the patient. (a) Rightward head impulses in the plane of the horizontal canal (RHHI) induce a correct response in terms of the gain of the VOR; fast eye movements in the contrary direction of the eye after the reflexive eye movement are seen as part of the spontaneous nystagmus and imbalanced vestibular nuclei function in the acute stage. (b) Leftward head impulses in the plane of the horizontal canal (LHHI) do not evoke a correct eye response; gain is very low and is followed by refixation saccades mixed with the fast phases of the spontaneous nystagmus.

*Patient 2.* A 22-year-old man suffered sudden vestibular damage in the context of his cholesteatomatous chronic otitis media. The fistula test (performed as above) was negative and the bedside head impulse test was positive. With the vHIT we were able to record a very low gain for head impulses towards the affected side with covert and overt refixation saccades as a response to the vestibular deficit and because of the spontaneous nystagmus beating towards the normal ear as well as anticompensatory quick eye movements for head movements towards the nonaffected side (Figure 2).

**Figure 3:**
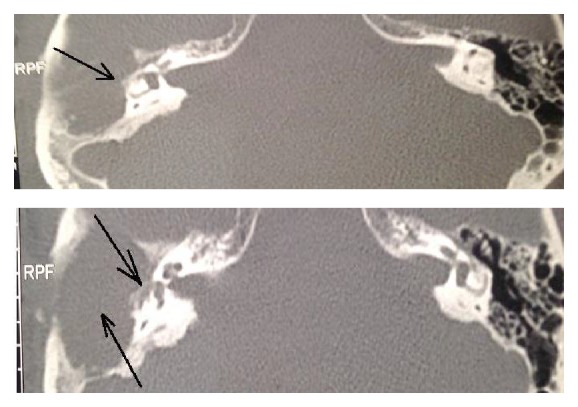
Case 3. Two different slices of the CT scan showing the fistula of the right horizontal semicircular canal and the middle ear filled with cholesteatoma and inflammatory tissue.

**Figure 4:**
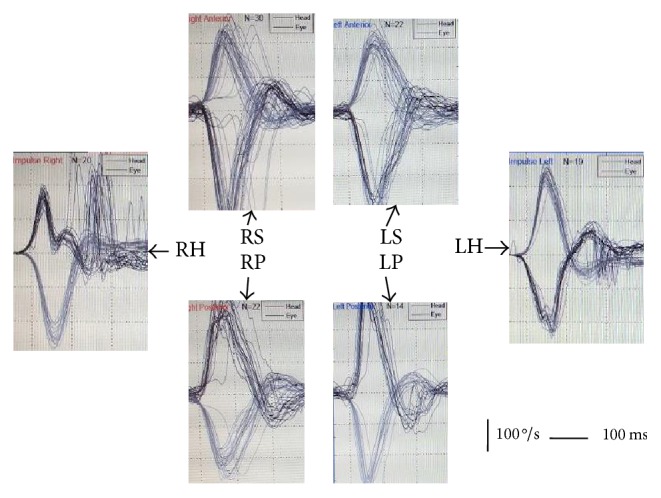
Case 3. Video head impulse test of the 6 semicircular canals. R (right) and left (L) ears, superior (S), horizontal (H), and posterior (P) canals. In the case of the affected canal (RH) the normal eye response is substituted by covert refixation saccades.

*Patient 3.* A 43-year-old patient presented with chronic otitis media with cholesteatoma that progressed over many years with hearing loss and otorrhea. As in patient 1 the progressive nature of the disorder led to adequate compensation. In this case, we were able to demonstrate that the damage was restricted to the area of the fistula (Figure 3) in the horizontal canal (Figure 4). The fistula test was negative and in the vHIT only the gain for head impulses towards the affected side in the plane of the horizontal semicircular canal was abnormal.

## 3. Discussion

In this report, we have demonstrated three patients with radiologically and surgically confirmed horizontal semicircular canal fistula but with a false negative fistula test. Bedside head impulse testing was able to show the abnormal vestibule-ocular reflex and in one case was able to localize the deterioration to a particular semicircular canal.

For the proper surgical approach in patients with extensive chronic otitis media with cholesteatoma it is important to ascertain whether or not the vestibular system is functioning, in particular when a fistula is suspected. With our work we confirm the finding that the FT is not sensitive enough in certain patient populations. This case series has demonstrated the utility of the head impulse test, performed at the patient's bedside with or without a video based system. This testing does not require an anatomically normal external and/or middle ear (as for the calorics), is faster, and lacks much of the side-effects the calorics and rotatory chair tests have. More importantly, it is a very specific test of the vestibuloocular reflex [8].

In patient 3 the vestibular deficit was localized specifically to the canal with the fistula. This novel finding was obtained due to the possibility of assessing all semicircular canals. As shown in otopathology records of patients with chronic otitis media with cholesteatoma, some degree of serous labyrinthitis close to the fistula can occur, without spread to other parts of the inner ear, whenever episodes of infection occur or are incorrectly treated [9]. This compartmentalization of the inner ear has been demonstrated before between the cochlea and vestibule [10] and within the vestibule between canals. This is the basis for their surgical occlusion in Benign Paroxysmal Positional Vertigo [11] or Superior Semicircular Dehiscence [12].

## 4. Conclusion

False negative fistula tests are not uncommon in patients with cholesteatoma of the middle ear. In this case series, we demonstrated that a localized or completely dead labyrinth can be easily diagnosed using the head impulse test, allowing for the proper surgical approach. We believe the head impulse test (bedside or vHIT) should be a component of the examination when performing fistula testing. The Lucae and Halmagyi-Curthoys signs were described 100 years apart now joined for the proper evaluation of patients with chronic otitis media.

## Figures and Tables

**Table 1:** Summary of findings for the three patients included in this study.

	Patient 1	Patient 2	Patient 3
Sex	Male	Male	Male

Age (years)	25	22	43

Diagnosis	Meningitis secondary to cholesteatoma	COM	COM

Ear affected	Left	Left	Right

Ear symptoms	Progressive hearing loss & Otorrhea	Progressive hearing loss & Otorrhea	Progressive hearing loss & Otorrhea

Clinical data	Meningitis	Acute vertigo; 1 week	No vertigo

Ear exam	Abundant otorrhea and cholesteatoma; no identification of middle ear structures. No vertigo on aspiration	Otorrhea and polyp filling the EAC	Otorrhea in the EAC; tympanic membrane perforation with cholesteatoma filling the middle ear

Audiometry	Right: normal	Right: normal	Right: moderate mixed hearing loss
	Left: cophosis	Left: profound mixed hearing loss	Left: normal

Radiology	Chol & Left horizontal canal fistula & Tegmen tympani dehiscence	Chol & Left horizontal canal fistula	Chol & Right horizontal canal fistula

Fistula test	Negative	Negative	Negative

Spontaneous nystagmus	Negative	Rightward	Negative

cHIT	Left positive	Left positive	Right positive

vHIT	n.d.	Grh: 0.77; Glh: 0.15	Gra = 1.2; Grh = 0.27; Grp = 1.1; Gla = 1.2: Glh = 0.8; Glp = 1.2

COM: cholesteatomatous otitis media; EAC: external auditory canal; Chol: suspected cholesteatoma in CT scan; cHIT: bedside head-impulse test. Positive if refixation saccades are seen after head impulses to one or the other side; vHIT: video head-impulse test; Gra: gain of the VOR for head impulses in the plane of the right superior semicircular canal; Grh: gain of the VOR for rightward head impulses; Grp: gain of the VOR for head impulses in the plane of the right posterior semicircular canal; Gla: gain of the VOR for head impulses in the plane of the left superior semicircular canal; Gl: gain of the VOR for leftward head impulses; Glp: gain of the VOR for head impulses in the plane of the left posterior semicircular canal; n.d.: not done.

## Additional Points

*Summary.* The cause of a false negative fistula test needs to be further evaluated for the proper surgical approach in middle ear surgery for patients with chronic otitis media. Vestibular function can be assessed independently of external and middle ear status with the head impulse test (bedside with or without and video assistance) and its information is complementary to that obtained by the pressure test. In the case of a negative fistula test and positive head impulse test, caution must still be exercised at the time of surgery unless information about the function of all the semicircular canals is available.
